# Impact of a multidisciplinary pain management team on acute and chronic pain management after total knee arthroplasty

**DOI:** 10.1007/s00540-025-03529-3

**Published:** 2025-06-28

**Authors:** Tomoo Yuba, Shunsuke Yamamoto, Hironobu Uematsu, Takeshi Yoshida

**Affiliations:** 1 Department of Anesthesiology and Intensive Care Medicine, Osaka University Graduate School of Medicine, Suita, Japan; 2 Department of Anesthesiology, Ikeda City Hospital, Ikeda, Japan

**Keywords:** Total knee arthroplasty, Multidisciplinary pain management team, Chronic postsurgical pain, Acute pain management

## Abstract

**Purpose:**

The impact of multidisciplinary team (MDT) interventions on chronic postsurgical pain remains unclear. This study evaluated the effects of MDT on acute and chronic pain outcomes in total knee arthroplasty (TKA) patients.

**Methods:**

This retrospective cohort study included 324 patients who underwent unilateral TKA between April 2017 and March 2023. The patients were divided into pre-MDT *(n* = 147) and post-MDT (*n* = 177) groups. The MDT, comprising anesthesiologists, pain nurses, and pharmacists, conducted daily rounds from postoperative day (POD) 1 to 4. The acute (duration of nerve block, incidence of breakthrough pain [BTP]) and chronic outcomes at 3 months (movement-related numerical rating scale [NRS] scores, regular analgesic use) were compared. Statistical significance was set at *p* < 0.05.

**Results:**

The preoperative demographics were comparable between groups. The MDT significantly prolonged nerve block duration (2.7 ± 1.0 vs. 3.0 ± 1.5 days, *p* = 0.027) and reduced BTP incidence (50.3 vs. 29.4%, *p* = 0.0001). At 3 months, movement-related NRS scores were lower in the post-MDT group (4.2 ± 3.5 vs. 2.1 ± 2.4, *p* = 0.025), while regular analgesic use showed no significant difference (21.8 vs. 16.9%, *p* = 0.31).

**Conclusion:**

MDT intervention improved acute pain management by reducing BTP and ensuring optimal nerve block use. Additionally, MDT was associated with better chronic pain outcomes, reflected in lower movement-related NRS scores at 3 months. These findings highlight MDTs’ role in improving acute pain management and reducing movement-related pain at 3 months after TKA.

## Introduction

Total knee arthroplasty (TKA) is one of the most commonly performed surgical procedures worldwide, particularly for treating advanced osteoarthritis and other degenerative joint disorders. This procedure serves as a definitive intervention to restore knee function, alleviate chronic pain, and enhance mobility, enabling patients to regain independence and participate in daily activities. With the aging global population and the increasing prevalence of osteoarthritis, the demand for TKA has increased dramatically. By 2030, the annual volume of TKA surgeries is projected to exceed 5 million procedures globally, highlighting the growing importance of optimizing perioperative care [1].

Postoperative pain management remains the cornerstone of successful TKA recovery. Effective postoperative pain management is crucial to enhance recovery and minimize complications. Inadequate pain control can lead to increased stress responses that may adversely affect wound healing and increase the risk of thromboembolic events [2]. Moreover, inadequate pain management can delay functional recovery, prolong rehabilitation, and negatively affect long-term surgical outcomes. Chronic postsurgical pain (CPSP), a debilitating condition characterized by pain persisting beyond the normal healing period, affects approximately 20% of patients after TKA [3]. CPSP can significantly impair quality of life, limit physical activity, and impose a substantial burden on healthcare systems [4].

The current best practices in TKA pain management emphasize multimodal analgesia, a strategy that combines pharmacological and non-pharmacological interventions to address different pain pathways. Among these, peripheral nerve blocks, such as femoral and sciatic nerve blocks, are widely regarded as the gold standard [5]. These techniques provide superior analgesia compared to single nerve blocks and facilitate early ambulation without the systemic side effects associated with opioids [6]. However, despite these advances, a significant proportion of patients experience breakthrough pain (BTP) or incomplete analgesia during their hospital stay, necessitating additional interventions. Inadequate maintenance or premature discontinuation of continuous nerve blocks has been associated with increased postoperative pain, higher opioid consumption, and delayed functional recovery in TKA patients [7]. Therefore, ensuring the appropriate duration of nerve block administration is crucial for optimizing acute pain management.

The implementation of a multidisciplinary pain management team (MDT) has emerged as a promising approach to address the limitations of traditional pain management. Comprising anesthesiologists, pain nurses, and pharmacists, MDTs offer a holistic, patient-centered approach by tailoring pain management strategies to individual needs. In surgical settings such as breast surgery, MDT interventions have demonstrated reductions in pain scores, analgesic consumption, and length of hospital stay, highlighting their potential to enhance perioperative care [8, 9]. However, evidence regarding the effect of MDTs on chronic outcomes, such as the prevention of CPSP, remains sparse.

A critical yet underexplored aspect of postoperative pain management is its influence on rehabilitation and long-term functional outcomes. Effective pain control facilitates early mobilization, which is a key determinant of successful rehabilitation. Conversely, poorly controlled pain can exacerbate fear of movement, delay functional recovery, and increase the risk of chronic pain development [10, 11]. However, the relationship between optimal analgesia, rehabilitation progress, and long-term outcomes in patients has yet to be fully elucidated.

This study aimed to address these gaps by evaluating the effect of MDT on both acute and chronic pain outcomes in patients who underwent TKA. Specifically, we examined how MDT interventions influenced key metrics such as nerve block duration, BTP incidence, and movement-related pain scores at 3 months, postoperatively. By bridging this knowledge gap, this study sought to provide robust evidence supporting the integration of MDTs into routine perioperative care for patients undergoing TKA.

## Methods

### Study design and population

This retrospective cohort study was conducted at Osaka University Hospital from April 2017 to March 2023. The patients who underwent unilateral TKA under general anesthesia, combined with both a continuous saphenous nerve block and a single-shot sciatic nerve block were included. Both nerve blocks were performed after the completion of surgery but before emergence from general anesthesia. Patients who underwent TKA for reasons other than osteoarthritis or bilateral TKA were excluded. Patients who underwent TKA for indications other than osteoarthritis (e.g., rheumatoid arthritis, post-traumatic arthritis) were excluded to minimize variability in preoperative pain mechanisms and to achieve a more homogeneous study population. In addition, since osteoarthritis was the most common indication for TKA at our institution during the study period, we focused our analysis on patients with osteoarthritis. This study included a total of 324 patients who underwent unilateral total knee arthroplasty (TKA) for osteoarthritis at Osaka University Hospital (147 in the pre-MDT group and 177 and in the post-MDT group). Ethical approval was obtained from the Osaka University Hospital Ethics Committee (Approval No. 21489) and conducted in accordance with the Declaration of Helsinki and its subsequent amendments. The requirement for written informed consent was waived by the ethics committee based on the opt-out methodology in accordance with national ethical guidelines.

### Pain assessment and definition of breakthrough pain

BTP was defined as the use of rescue analgesics (loxoprofen or acetaminophen) as required for postoperative pain. The postoperative pain typically peaks within the first three to four days following surgery; therefore, we defined the tracking period for BTP as up to postoperative day (POD) 4 to align with this timeline [12–14].

### Nerve block techniques

To optimize postoperative pain management, all patients received nerve blocks prior to emergence from general anesthesia. A continuous saphenous nerve block was performed under ultrasound guidance with the placement of a catheter for continuous infusion of local anesthetic, targeting the saphenous nerve in the mid-thigh region. For the sciatic nerve block, a single-shot injection of local anesthetic was administered under ultrasound guidance, targeting the sciatic nerve approximately 10 cm proximal to the knee joint. To standardize the quality of nerve blocks, our institution holds regular internal workshops where anesthesiologists are trained in ultrasound-guided techniques using anatomical models. These blocks are performed under supervision by instructors experienced in peripheral nerve blocks. The continuous blocks used 0.167% ropivacaine at an initial infusion rate of 4 mL/h, while single-shot sciatic nerve blocks were also performed with ropivacaine. The duration of the continuous nerve block was defined as the number of postoperative days until catheter removal, as documented in the electronic medical record. No objective sensory or motor block assessment was routinely recorded beyond the initial postoperative period.

### Data collection

The data were extracted from electronic medical records and included patient demographics (age, sex, height, weight, and body mass index [BMI]) and postoperative outcomes such as BTP requiring additional analgesia, rehabilitation start times, duration of nerve block, length of hospital stay, and numerical rating scale (NRS) scores at 3 months postoperatively. Additionally, intraoperative opioid consumption was recorded. At our institution, remifentanil was continuously infused during surgery, and fentanyl was administered as a single bolus. Therefore, intraoperative fentanyl dose (μg) was used as the measure of intraoperative opioid consumption. In our institution, postoperative rehabilitation is typically initiated after the surgical drain has been removed, provided that the patient’s vital signs are stable and no major complications are present. Discharge is generally permitted once patients are able to ambulate safely with a walking cane and have achieved basic activities of daily living.

### Multidisciplinary pain team composition

The MDT comprised anesthesiologists, pain nurses, and pharmacists. At least one member from each specialty attended the postoperative rounds. The MDT followed a structured pain evaluation protocol (Table 1) and intervened when necessary to optimize pain control. The rounds were conducted daily from POD 1 to 4, with at least one visit per day. When postoperative pain control was deemed insufficient, the MDT took several actions tailored to each patient’s needs. These included instructing medical staff to increase the basal infusion rate or activate the patient-controlled bolus of the continuous nerve block, and adjusting systemic analgesics. If catheter migration or poor block efficacy was suspected, the catheter was removed and analgesia was switched to an alternative regimen, such as scheduled administration of acetaminophen or NSAIDs. These interventions were implemented based on direct patient assessments during daily rounds.

**Table 1:**
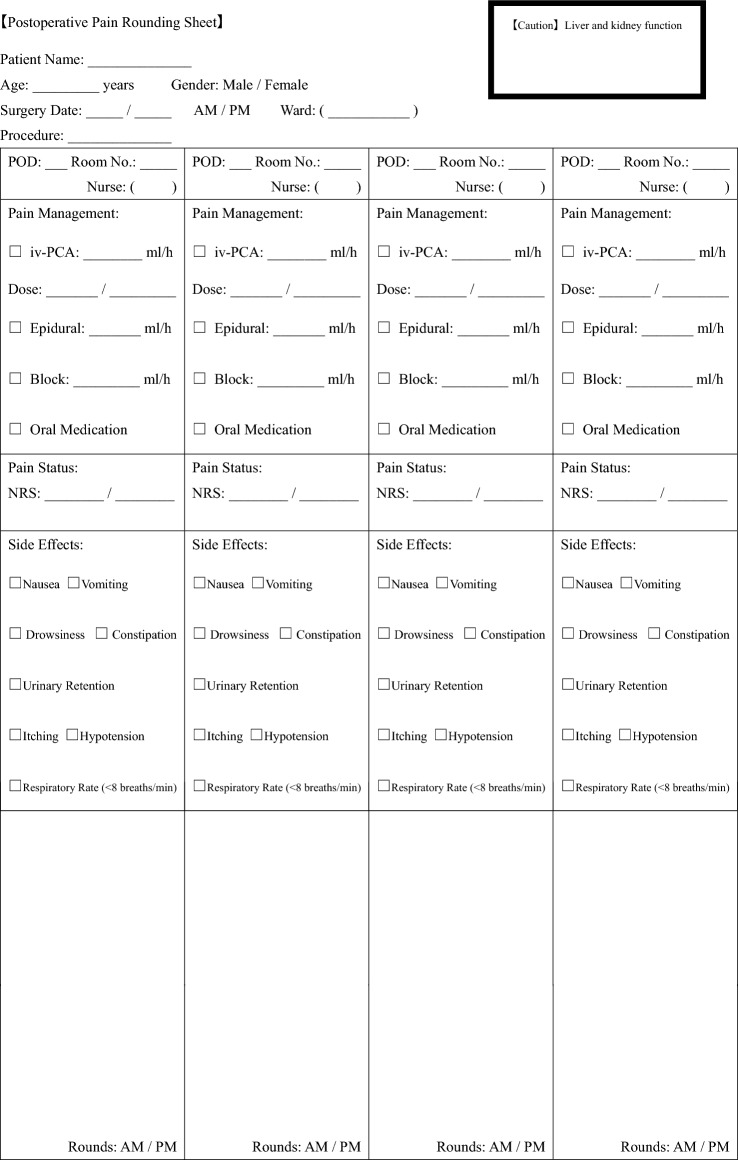
Multidisciplinary pain management team (MDT) evaluation sheet

This table presents the structured evaluation sheet used by the MDT at the Osaka University Hospital. The sheet captures pain management metrics, analgesic usage, and rehabilitation progress during the postoperative rounds.

### Statistical analysis

Data are expressed as the mean ± standard deviation or percentages. Continuous variables were compared using the student’s *t*-test or Mann–Whitney *U* test, and categorical variables were analyzed using the chi-square test or Fisher’s exact test. Statistical significance was set at *p* < 0.05.

## Results

### Patient demographics

There were no statistically significant differences in the preoperative characteristics, including age, sex, height, weight, or BMI, between the pre- and post-MDT groups (Table 2).

**Table 2:** Preoperative patient characteristics before and after multidisciplinary pain management team (MDT) implementation

	Before MDT initiation (*n* = 147)	After MDT initiation (*n* = 177)	*p*-value
*Preoperative information*			
Age (years)	73.1 ± 8.8	74.3 ± 10.3	0.28
Gender(female)	115 (78.2%)	150 (84.7%)	0.13
Height (cm)	153.8 ± 8.5	153.4 ± 8.4	0.63
Weight (kg)	60.6 ± 11.8	60.4 ± 11.0	0.85
BMI	25.5 ± 3.9	25.7 ± 4.4	0.81

This table compares the preoperative characteristics of the patients treated before (*n* = 147) and after (*n* = 177) MDT implementation. No significant differences were observed in age, sex, height, weight, or body mass index (BMI, a measure of body fat based on height and weight)

### Acute postoperative outcomes

**Fig. 1:**
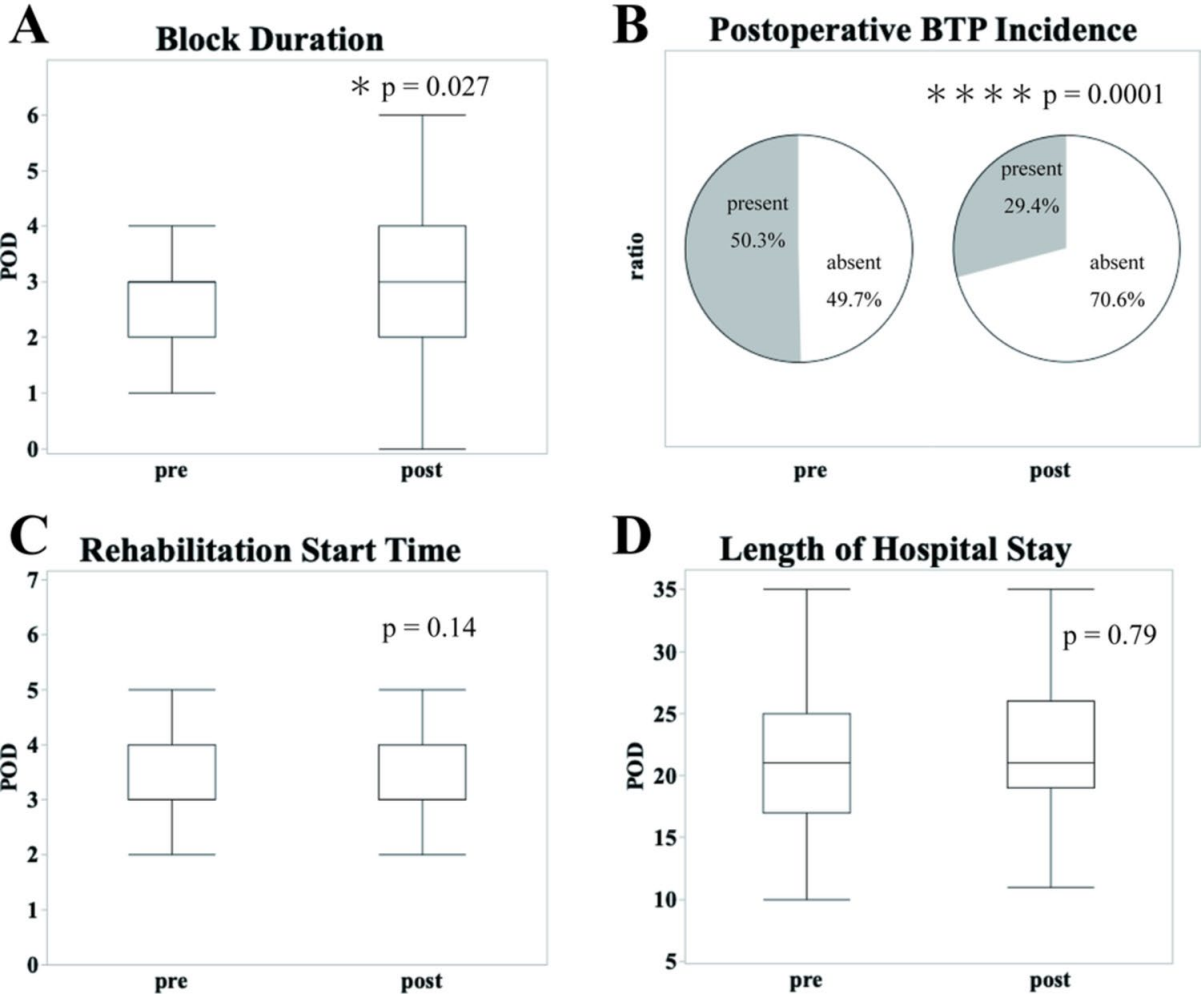
Acute postoperative outcomes with and without multidisciplinary pain management team (MDT) intervention. This figure shows the acute postoperative outcomes. **A** duration of nerve block, **B** incidence of breakthrough pain (BTP), **C** rehabilitation start time, **D** length of hospital stay. The MDT group demonstrated a longer nerve block duration and reduced BTP

The MDT intervention was associated with a significant increase in the duration of continuous nerve blocks (2.7 ± 1.0 days in the pre-MDT group vs. 3.0 ± 1.5 days in the post-MDT group, *p* = 0.027). Additionally, the frequency of BTP requiring additional analgesics was significantly lower in the post-MDT group than in the pre-MDT group (50.3 vs. 29.4%, *p* = 0.0001) (Fig. 1; Table 3). However, no significant differences were observed in the timing of rehabilitation initiation (3.5 ± 0.7 days in the pre-MDT group vs. 3.4 ± 0.6 days in the post-MDT group, *p* = 0.14) or the length of hospital stay (22.2 ± 10.7 days in the pre-MDT group vs. 22.5 ± 6.6 days in the post-MDT group, *p* = 0.79) (Fig. 1, Table 3). Intraoperative fentanyl consumption was comparable between groups (pre-MDT: 252.3 ± 107.7 μg vs. post-MDT: 272.7 ± 96.3 μg, *p* = 0.33).

**Table 3:** Comparison of acute and chronic postoperative outcomes before and after multidisciplinary pain management team implementation

	Before MDT initiation (*n* = 147)	After MDT initiation (*n* = 177)	*p*-value
*Acute postoperative information*			
Block duration (days)	2.7 ± 1.0	3.0 ± 1.5	0.027
Postoperative BTP Incidence (%)	74 (50.3%)	52 (29.4%)	0.0001
Rehabilitation start time (days)	3.5 ± 0.7	3.4 ± 0.6	0.14
Length of hospital stay (days)	22.2 ± 10.7	22.5 ± 6.6	0.79
*chronic postoperative information*			
Resting NRS	2.2 ± 2.8	1.1 ± 1.7	0.13
Movement NRS	4.2 ± 3.5	2.1 ± 2.4	0.025
Regular analgesic use at 3 months Post-Op	32 (21.8%)	30 (16.9%)	0.31

This table summarizes the key postoperative outcomes, including nerve block duration, incidence of breakthrough pain, and numerical rating scale (NRS) scores. Significant improvements were observed in the acute outcomes, whereas the chronic outcomes showed significant differences in the movement-related NRS scores

### Chronic postoperative outcomes

**Fig. 2:**
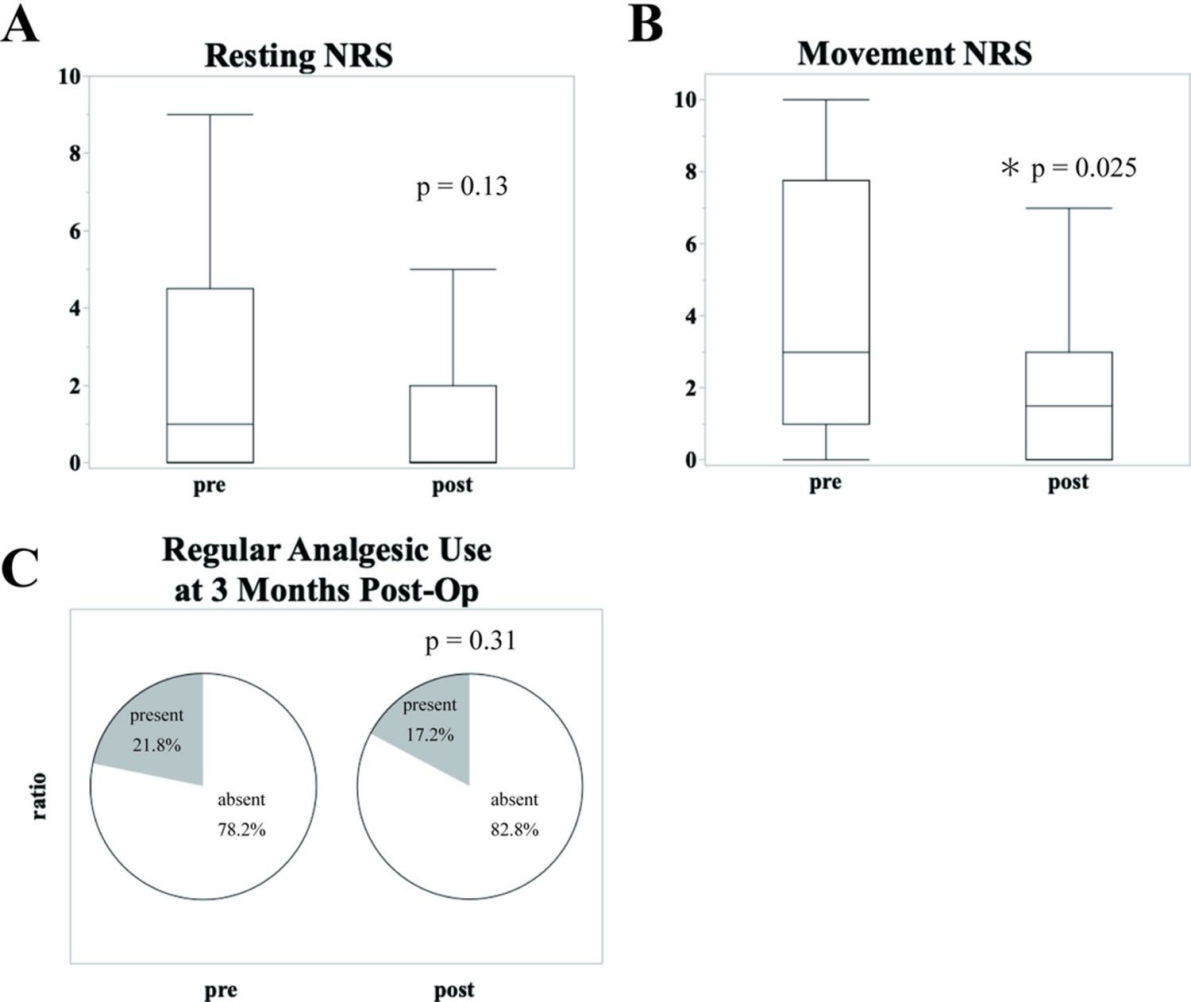
Chronic postoperative outcomes at 3 months with and without pain management team (MDT) intervention This figure presents the chronic postoperative outcomes at 3 months, including **A** regular analgesic use, **B** resting numerical rating scale (NRS) scores; and **C** movement-related NRS scores. Significant reductions in the movement-related NRS scores were observed in the MDT group

At 3 months postoperatively, the patients in the MDT group had significantly lower movement-related NRS scores (4.2 ± 3.5 in the pre-MDT group vs. 2.1 ± 2.4 in the post-MDT group, *p* = 0.025). However, no significant differences were observed in resting NRS scores (2.2 ± 2.8 in the pre-MDT group vs. 1.1 ± 1.7 in the post-MDT group, *p* = 0.13) or the proportion of patients requiring regular analgesic use (21.8% in the pre-MDT group vs. 16.9% in the post-MDT group, *p* = 0.31) (Fig. 2; Table 3).

## Discussion

The results of this study demonstrate that the presence of an MDT significantly improved both acute and chronic postoperative outcomes in patients undergoing TKA. Importantly, there were no notable changes in surgical techniques and perioperative care protocols other than the implementation of the MDT during the study period. The surgical procedures and anesthetic practices remained consistent across both groups. Therefore, the observed improvements in postoperative pain are unlikely to be attributed to advances in other perioperative practices. MDT intervention was associated with a reduction in BTP and longer duration of continuous nerve block, which contributed to better acute pain control.

### Acute pain management

The extension of the continuous nerve block duration in the MDT group likely contributed to the lower incidence of BTP. Additionally, the active involvement of the MDT in instructing both patients and medical staff on the proper use and management of nerve block catheters played a significant role in achieving these improved outcomes. In addition, the absence of significant differences in intraoperative fentanyl consumption between groups suggests that the observed improvements in acute postoperative pain were likely attributable to MDT intervention rather than differences in intraoperative opioid management. This aligns with existing literature emphasizing the importance of multimodal analgesia in reducing acute postoperative pain and facilitating early rehabilitation [10, 11]. Inadequate pain management after TKA can hinder rehabilitation, increase morbidity and mortality rates, decrease patient satisfaction, and lead to persistent CPSP [15]. By minimizing BTP, the MDT enables patients to engage in early mobilization, which is critical for optimizing functional recovery after TKA. However, no significant differences were observed in the rehabilitation start times or the length of hospital stay. Although a study on postoperative pain management following breast surgery reported a reduction in the hospital stay with an MDT intervention [8], this finding did not align with our results. The lack of difference in rehabilitation start times may be attributable to institutional factors. At our hospital, rehabilitation services are not initiated on weekends or public holidays. Consequently, the patients undergoing surgery later in the week often experience delays in initiating rehabilitation, which could have influenced this outcome. Moreover, the continuous saphenous nerve block and single-shot sciatic nerve block techniques employed in this study may represent highly effective perioperative management strategies for TKA [5]. These methods likely facilitated early rehabilitation even before the MDT intervention, which could explain why there was no significant difference in the timing of rehabilitation initiation between the pre- and post-MDT groups. Notably, as demonstrated in this study, targeting distal sites far from the central nerve pathways reduces the risk of muscle weakness and falls, thereby minimizing barriers to early rehabilitation [16]. Post-TKA rehabilitation is critical for achieving optimal functional recovery and improving patient outcomes [17]. As demonstrated in this study, the combination of effective pain control through nerve blocks and MDT may significantly enhance postoperative rehabilitation and facilitate better recovery.

### Chronic pain management

At 3 months postoperatively, patients in the MDT group reported significantly lower NRS scores during movement, suggesting that early intervention with MDT contributed to reduced chronic pain. This finding supports the hypothesis that effective acute pain management is essential for preventing the transition to chronic pain [4]. Pain persisting for several weeks to 3 months postoperatively is a strong predictor of subsequent CPSP [18]. The observed reduction in pain 3 months after the MDT intervention suggests that MDTs may play a significant role in preventing the transition to chronic pain. However, further research is needed to evaluate the effectiveness of MDTs in long-term pain control beyond the 3-month postoperative period. However, it is important to acknowledge that chronic pain is influenced by a multitude of factors beyond acute pain management. Variables such as patient activity levels, weight changes, lifestyle habits, and psychological state are likely to play significant roles in the development and persistence of chronic pain; however, these factors were not evaluated in the present study. This limitation leaves some uncertainty regarding the specific mechanisms driving the observed reduction in the NRS scores. Although there was no significant difference in the percentage of patients requiring regular analgesics, the reduction in NRS scores indicated that MDT interventions may help mitigate movement-related discomfort, thereby improving patients’ quality of life. Notably, the prescription of regular analgesics, including their type and timing, was not standardized across the study population. These decisions were left to the discretion of the individual outpatient physicians. Although fewer patients in the MDT group required regular analgesic use, attributing this entirely to MDT interventions preventing the transition to chronic pain warrants further investigation and caution. These nuances highlight the complexity of chronic pain management and the need for more comprehensive assessments in future studies.

### Multidisciplinary team’s role

The role of MDT in improving pain outcomes extends beyond pharmacological intervention. At our institution, MDT involvement begins before surgery during the preoperative anesthesia consultation. At this stage, the team introduces themselves and provides detailed explanations of the perioperative pain management strategies. This proactive approach may offer substantial reassurance to patients, alleviate anxiety about upcoming surgery, and foster positive expectations about perioperative pain management outcomes. Additionally, as previously noted, the MDT interventions are not limited to direct patient care but also encompass education and guidance for ward medical staff, including primary physicians and nurses. The training sessions were held to equip the staff with the skills necessary for catheter management, including techniques for securing catheters, identifying and addressing complications such as dislodgement or infection, and monitoring pain scores. Nurses were trained to perform regular catheter inspections and ensure proper hygiene protocols, whereas doctors were instructed on the criteria for continuing or discontinuing nerve block therapy. These efforts will likely enhance the overall quality of care, ensuring that both patients and staff are better equipped to effectively address postoperative pain. Collaboration among anesthesiologists, pain nurses, and pharmacists within the MDT facilitates seamless communication and timely adjustments in pain management strategies. Multidisciplinary pain management, involving surgeons, anesthesiologists, nurses, physical therapists, and pharmacists, plays a critical role in the care of patients undergoing TKA. The importance of such comprehensive team-based approaches has been increasingly emphasized in the context of postoperative management [19]. Comprehensive multidisciplinary management of postoperative pain not only alleviates patient discomfort but also contributes to shorter hospital stays, decreased healthcare costs, and greater patient satisfaction [20]. This interdisciplinary approach aligns with the positive outcomes reported in other studies involving MDT interventions in various surgical settings [8, 21]. The combined effects of patient education, staff training, and multidisciplinary collaboration underscore the importance of MDT in achieving optimal pain control and improving recovery outcomes. The training and development of nurses specializing in pain management could play a pivotal role in improving patient outcomes [22]. Such expertise enhances the quality of pain management and highlights the potential impact of specialized nursing care in MDT interventions. The broader adoption of MDTs and integration of pain management-trained nurses should be prioritized in the future. The results of this study highlight the importance of MDT interventions for both acute and chronic pain management after TKA. Multidisciplinary approaches involving continuous nerve blocks, timely pharmacological interventions, and patient education can significantly improve postoperative recovery and reduce the risk of chronic pain. Although the introduction of an MDT did not significantly shorten the length of hospital stay, the observed reduction in chronic postoperative pain, particularly in the frequency of regular analgesic use, may offer important long-term economic advantages. Chronic pain is known to cause substantial societal costs through decreased productivity, increased healthcare utilization, and diminished quality of life. Even in the absence of statistically significant reductions in analgesic use, the trend observed in our study suggests a potential benefit in reducing the long-term burden of chronic pain, thereby justifying the initial personnel investment associated with MDT implementation. The implementation of MDTs in pain management should be considered standard practice, especially for major surgeries such as TKA.

### Study limitations

This study had several limitations that warrant discussion. First, the retrospective design may have introduced selection bias, as data were collected from existing medical records without the ability to control for all confounding variables. Second, the tracking of BTP was limited to the initial four PODs, corresponding to the period of MDT rounds. However, the postoperative pain trajectories often vary beyond this timeframe, and additional follow-ups could offer deeper insights into BTP management. Furthermore, while MDT interventions have shown the potential for reducing chronic pain, other factors contributing to chronic pain, such as patient activity levels, weight changes, lifestyle habits, and mental health, were not assessed. These unmeasured variables may have influenced the observed outcomes and require further investigation. Additionally, the regular use of analgesics was based on prescriptions that varied among outpatient providers, with no standardized protocol for the type or timing of analgesics. Finally, the study was conducted at a single institution with a specific MDT structure and perioperative protocol, which may limit the generalizability of the findings to other settings. Future studies should validate these results, standardize analgesic protocols, and evaluate the broad implications of MDT interventions in various clinical contexts.

## Conclusion

The introduction of an MDT significantly improved the acute and chronic postoperative pain outcomes in patients who underwent TKA. By reducing BTP, ensuring the appropriate maintenance of nerve blocks, and lowering NRS scores during movement, MDT can enhance both pain control and recovery. Further research is necessary to assess the applicability of MDT interventions in other surgical populations and explore their potential for broader healthcare improvements.

## Data Availability

All relevant data are within the paper and its Supplementary Information files.
